# Analysis of the prion protein gene in multiple system atrophy

**DOI:** 10.1016/j.neurobiolaging.2016.09.021

**Published:** 2017-01

**Authors:** Viorica Chelban, Andreea Manole, Lasse Pihlstrøm, Lucia Schottlaender, Stephanie Efthymiou, Emer OConnor, Wassilios G. Meissner, Janice L. Holton, Henry Houlden

**Affiliations:** a Department of Molecular Neuroscience, UCL Institute of Neurology, London, UK; b National Hospital for Neurology and Neurosurgery, Queen Square, London, UK; c Department of Neurology, Medical and Pharmaceutical State University N. Testemitanu, Chisinau, Moldova; d Institute of Clinical Medicine, University of Oslo, Oslo, Norway; e Univ. de Bordeaux, Institut des Maladies Neurodégénératives, UMR 5293, Bordeaux, France; f CNRS, Institut des Maladies Neurodégénératives, UMR 5293, Bordeaux, France; g Centre de référence atrophie multisystématisée, CHU de Bordeaux, Bordeaux, France; h Reta Lila Weston Institute of Neurological Studies and Queen Square Brain Bank for Neurological Disorders, London, UK

**Keywords:** Multiple system atrophy, *PRNP*, Prion disease, Prion protein, Sporadic Creutzfeld-Jakob disease, α-synuclein, α-syn, confidence intervals, CI, cytoplasmic inclusions, GCIs, multiple system atrophy, MSA, sporadic Creutzfeld-Jakob disease, sCJD, odds ratio, OR, prion protein, PrP

## Abstract

Neurodegenerative diseases are a very diverse group of disorders but they share some common mechanisms such as abnormally misfolded proteins with prion-like propagation and aggregation. Creutzfeldt-Jakob disease (CJD) is the most prevalent prion disease in humans. In the sporadic form of CJD the only known risk factor is the codon 129 polymorphism. Recent reports suggested that α-synuclein in multiple system atrophy (MSA) has similar pathogenic mechanisms as the prion protein. Here we present 1 Italian family with MSA and prion disease. Also, cases of concurrent MSA and prion pathology in the same individual or family suggest the possibility of molecular interaction between prion protein and α-synuclein in the process of protein accumulation and neurodegeneration, warranting further investigations. We assessed the *PRNP* gene by whole-exome sequencing in 264 pathologically confirmed MSA cases and 462 healthy controls to determine whether the 2 diseases share similar risk factors. We then analyzed codon 129 polymorphism by Sanger sequencing and compared with previously published results in sporadic CJD. Homozygosity at codon 129 was present in 50% of pathologically confirmed MSA cases and in 58% of normal controls (odds ratio, 0.7 (95% confidence interval of 0.5–0.9)) compared with 88.2% in sporadic CJD. Our data show that the homozygous state of position 129 in the *PRNP* is not a risk factor for MSA. No other variants in the *PRNP* gene were associated with increased risk for MSA.

## 1 Introduction

Neurodegenerative diseases represent a significant cause of disability and life span reduction worldwide (Bird et al., 2003, Huisman et al., 2011, Pringsheim et al., 2014). They are a very diverse group with a wide variety of symptoms, but they share some common pathogenic mechanisms such as abnormally misfolded proteins that accumulate in neurons or glia. A second common hallmark of many neurodegenerative diseases is the prion-like mechanism of propagation and aggregation of these misfolded proteins.

Prion diseases are a group of fatal neurodegenerative conditions caused by the accumulation of abnormally folded prion protein (PrP). They can be classified as acquired, inherited, or sporadic. Sporadic Creutzfeld-Jakob disease (sCJD) is the commonest human prion disease.

Multiple system atrophy (MSA) is a rapidly progressing neurodegenerative disorder with late onset and poor prognosis. Although the exact mechanisms behind MSA are not entirely elucidated; the neuropathological hallmark of the disease is the accumulation of abnormally folded α-synuclein (α-syn) and the pathognomonic formation of glial cytoplasmic inclusions (Ahmed et al., 2012, Wenning et al., 2008). MSA is mainly a sporadic disease but a few familial cases with Mendelian inheritance have been reported (Hara et al., 2007, Wullner et al., 2004). However, no causal gene was identified in those families.

Recently, data have emerged suggesting mechanistic similarities between prion disease PrP and MSA α-syn protein misfolding, propagation and self-aggregation (Woerman et al., 2015), raising the question that these disorders might involve joint risk factors. So far, the only risk factor associated with sCJD is older age and a polymorphism in the host's prion gene locus *PRNP* at codon 129 (rs1799990); results confirmed in a Genome Wide Association Study (Mead et al., 2012). Unique to humans, this polymorphic site can either encode a methionine (M) or a valine (V) resulting in 3 possible genotypes: M129M, M129V and V129V. A large study including 300 patients showed that almost 90% of all sCJD cases were homozygous at codon 129 with the largest group presenting the M129M genotype (Parchi et al., 1999). For sCJD, the heterozygous state M129V appears to be protective against the disease, whereas the homozygous M129M and V129V genotypes carry increased risk for all forms of prion diseases suggesting that the genotype of *PRNP* at codon 129 influences the susceptibility and the phenotype of the sCJD (Mead et al., 2012). Recent data also confirm that the M/V 129 residue has a crucial role in prion protein aggregation (Skora et al., 2013).

The 129 codon *PRNP* polymorphism status has not been previously assessed in large MSA studies but was reported in a case of a 64-year-old patient, who developed sporadic prion disease 4 years after being diagnosed with MSA. The genotype in this case was M129M homozygous for *PRNP* (Shibao et al., 2008).

Here, we report an unusual co-occurrence of prion disease and MSA in 1 Italian family. In a pedigree of genetically confirmed Gerstmann-Sträussler-Scheinker syndrome, MSA developed in an individual negative for the pathogenic mutation. Given the recent reports of prion-like mechanisms in MSA and cases of associated MSA and prion pathology, we conducted a study to assess whether variants in *PRNP* and in particular the polymorphism status at codon 129 of *PRNP* gene represents a risk factor for MSA.

## 2 Methods

### 2.1 Subjects and genetic analysis

We have investigated by whole-exome sequencing that 264 pathologically confirmed MSA cases of Caucasian ethnicity from MSA Brain Bank and DNA Collaboration (see Acknowledgements). MSA-C, MSA-P, and combined MSA-P/C cases were included. DNA was extracted from brain tissue using a standard technique followed by next generation sequencing (Singleton et al., 2010). Whole-exome sequencing data were obtained from 462 healthy controls of Caucasian origin older than 65 who were confirmed postmortem to be neuropathologically normal from the Healthy Exomes database. Quality control of exome data included removal of samples with high missingness rate, excess heterozygosity, sex check failure, or evidence of cryptic relatedness. To assess ancestry from exome data, we identified a set of pruned, common SNPs (minor allele frequency >0.05), and extracted the same SNPs from all populations in 1000 Genomes Project phase 3 data. We then merged MSA, control, and 1000 Genomes data and performed principal component analysis, using the software package PLINK v.1.9. The first, second, and third principal components were used to visualize ancestry in plots and outliers were removed, leaving only samples that clustered together with the Caucasians in the final data set.

We also investigated by Sanger sequencing the *PRNP* 129 polymorphism in 238 of the pathologically confirmed MSA cases. *PRNP* PCR primers designed to span the region of interest (M129V polymorphism, rs# cluster id 1799990) and about 50 bp of flanking coding sequence (5′CTGGGGTCAAGGAGGTGG 3′and 5′ AACGGTGCATGTTTTCACGA 3′). The variant was PCR amplified using intronic primers. The purified PCR product was sequenced bi-directionally with Big Dye Terminator Kit v.3.1 (Applied Biosystems). Sanger sequencing was performed using a conventional protocol described elsewhere (Houlden et al., 2001). *PRNP* variant position is based on NCBI reference sequences: NM_000311, NP_000302 (www.ncbi.nlm.nih.gov).

Brain tissue obtained from QSBB was donated for research using ethically approved protocols and stored under a license from the Human Tissue Authority. DNA was extracted and investigated under approval of the joint ethics committee of UCL Institute of Neurology and the National Hospital for Neurology and Neurosurgery, London, UK (UCLH: 04/N034).

We investigated in our MSA clinic one family with both MSA and prion pathology.

### 2.2 Statistical analysis

Odds ratios and 95% confidence intervals were calculated to assess the association between *PRNP* variant in MSA versus control and MSA versus sCJD. We used published genotype data for the sCJD from a cohort of 300 cases (Parchi et al., 1999). The χ^2^ test was used to assess *p* value; 2-tailed *p* ≤ 0.05 was the significance level used for statistical analysis. We used the SPSS software for statistical analysis.

## 3 Results

### 3.1 *PRNP* genotype in MSA cases

After sequencing the whole *PRNP* gene we found no pathogenic mutation in our MSA cases. There were 4 coding variants in the *PRNP* with codon 129 polymorphism being the most frequent (Table 1).

Detailed analysis of the codon 129 polymorphism by Sanger sequencing showed that 129 homozygous variant was present in 50% of the 238 pathologically confirmed MSA cases (119 cases, 37.82% of M129M and 12.18% of V129V). The comparable figures were 58% in normal controls (OR 0.7; 95% CI of 0.5–0.9) and 88.2% in sCJD (OR 0.13 and *p* < 0.0001; Table 2).

### 3.2 Prion and MSA family

**Fig. 1:**
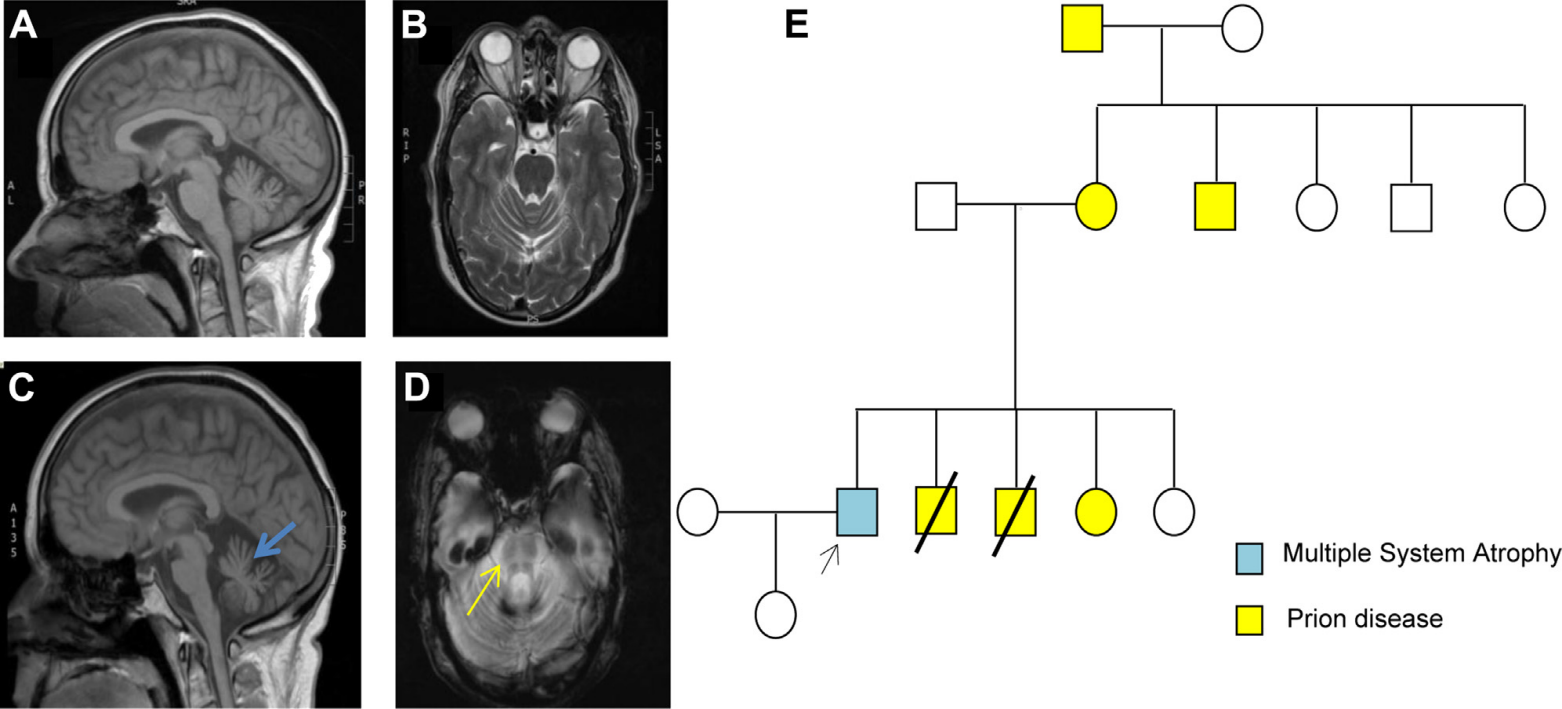
MRI results for the proband. (A, B): T1 sagittal and T2 transversal view in 2008 at onset of MSA symptoms. Only mild cerebellar atrophy was noted. (C, D): sagittal and transversal views 3 years later. Cerebellar atrophy (blue arrow) had progressed and additional brainstem atrophy with the typical “hot-cross bun sign” had appeared (yellow arrow). E. Pedigree demonstrating prion and MSA diseases in the family. The prion-affected cases are represented in yellow; the MSA-affected cases are represented in blue. Abbreviation: MSA, multiple system atrophy.

The family is originally from Sicily. The male proband is the eldest of a siblingship of 5 from nonconsanguineous parents. Their mother, a maternal uncle, maternal grandfather, 2 of his brothers, and 1 sister have been diagnosed with the hereditary prion disease Gerstmann-Sträussler-Scheinker syndrome caused by P102L mutation (Fig. 1E). His deceased father was diagnosed with Parkinsonism later in life but no final diagnosis was reached at the time. The proband started developing cerebellar ataxia at the age of 55. The symptoms progressed rapidly and 2 years later, the neurological examination revealed progressive ataxia, dysarthria, jerky pursuit, and hypometric saccades with downbeat nystagmus, dysphagia, mild bradykinesia, increased tone in the lower limbs, neurogenic bladder and bowel, autonomic failure and REM sleep behavior disorder. He had a stooped posture with small steps, shuffling gait and poor arm swing. A levodopa trial made his balance and orthostatic symptoms worse. Brain MRI conducted 3 years after disease onset showed marked global cerebellar and brainstem atrophy with cross-pontine signal change (Fig. 1C and D). FDG PET of brain and body scan showed cerebellar hypometabolism. This patient was negative for the familial P102L mutation, SCA panel genes, and FXTAS. He was diagnosed clinically with MSA.

## 4 Discussion

In this study, we assessed the *PRNP* genotype with a focus on the polymorphism status at codon 129 of *PRNP* gene as a risk factor for MSA in 264 pathologically confirmed MSA cases. Given the recent reports suggesting that α-syn of MSA has similar pathogenic mechanisms to the prion PrP in protein misfolding, propagation, and self-aggregation (Woerman et al., 2015), we assessed whether they share the same risk factor.

A previous study of 300 cases of sCJD found that M129V polymorphism of *PRNP* was central in determining the susceptibility and pathologic phenotype in sCJD (Parchi et al., 1999). More specifically, heterozygosity at codon 129 is protective against prion disease (Mead et al., 2012). It is generally believed that the protective effect of *PRNP* codon 129 heterozygosity relates to the fact that misfolded PrP propagation is most effective when the interacting proteins have identical primary structure (Collinge, 2005, Palmer et al., 1991). Hypothetically, an analogous situation might exist in synucleinopathies given the existence of common sequence polymorphisms in the amino acid sequence of α-synuclein, but coding variants in *SNCA* are very rare, and associated with autosomal dominant Parkinson's disease (Petrucci et al., 2016). The co-occurrence of prion disease and MSA, as reported earlier in a single individual (Shibao et al., 2008) and here in different members of a Sicilian family, might suggest the possibility of molecular interaction between PrP and α-synuclein in the process of protein accumulation and neurodegeneration, warranting investigation of the PRNP codon *129* variant in MSA.

Our data show a similar frequency of the heterozygous state M129V in MSA pathologically confirmed cases compared with the healthy controls. In contrast to what was previously shown for sCJD, it appears that the heterozygous state of M129V does not provide protective effects in MSA, although a larger study would be required to unequivocally determine this.

In many genetic association studies, accurate phenotyping is a major weakness, particularly when contrasted with the definitive nature of genotyping (Shibao et al., 2008). In our study, we have used pathologically confirmed MSA cases and matched normal pathologically confirmed cases to ensure accuracy of results.

In conclusion, this is the first large study assessing the *PRNP* gene and in particular the polymorphism status at codon 129 of *PRNP* gene as a risk factor for MSA. Our data show that MSA does not share the same *PRNP* 129 locus as a risk factor.

## Disclosure statement

The authors have no conflicts of interest to disclose.

## Figures and Tables

**Table 1:** *PRNP* coding variants found in our pathologically confirmed MSA cohort

Variant	Type of mutation	Status	Total nonreference alleles	Total observed alleles	Allele frequency	OR	P
rs138688873 c.246_269delACAGCCTCATGGTGGTGGCTGGGG p.Pro84_Gln91del	inframe deletion	MSA	3	525	0.0057	0.74	0.54
		Controls	7	913	0.0076		
rs201423990 c.372C>G	synonymous	MSA	2	424	0.0047	1.08	NA
		Controls	4	920	0.0043		
rs1799990 c.385A>G p.M129V	missense	MSA	200	528	0.38	0.7	0.04
		Controls	295	924	0.32		
rs150351644 c.424G>A p.G142S	missense	MSA	1	528	0.0019	NA	NA
		Controls	0	924	0		

Key: OR, odds ratio; MSA, multiple system atrophy.

Total observed alleles-calculated as 2 chromosomes per individual.

**Table 2:** *PRNP* codon 129 genotype distributions within our multiple system atrophy (MSA) group, healthy controls, and sporadic Creutzfeld-Jakob disease (sCJD)

Genotype	MSA	Controls	sCJD^a^	MSA vs controls	
				Odds ratio (95% CI)	*p* value
M129V heterozygous	119 (50%)	191 (41.4%)	35 (11.8%)	0.7 (0.5–0.9)	*p* = 0.03
M129M and V129V homozygous	119 (50%)	271 (58.6%)	265 (88.2%)		

Key: CI, confidence interval.

a - (Parchi et al., 1999).
